# 

**Published:** 1902-06

**Authors:** 


### Society Notes.

### The North Texas District Medical Society.

The North Texas District Medical Society will hold its regular semi-annual meeting at Fort Worth, June 17th, 18th, 19th, inst. Excursion rates. Program not received in time for publication. A big meeting is expected. Splendid hotel accommodations; special rates to doctors.

### The Austin District Medical Society.

The Austin District Medical Society will hold its regular semi-annual meeting at Austin, Texas, Thursday and Friday, June 12th and 13th, inst., immediately after the closing of commencement exercises at the University. Excursion rates for the commencement hold good for this meeting, covering the dates above given. Let there be a full muster of the clans to discuss the proposed reorganization all over the State. General Clinic at the Seton Infirmary.

Dr. F. R. Collard, of Wheelock, was elected President of the Brazos Valley Medical Association at the Marlin Meeting. Next meeting will be at Rockdale, second Tuesday in November.

The East Texas Medico-Chirurgical Society held its regular semi-annual meeting at Palestine, May 28th-29th, ult. We were not able to be present, and have not to date of going to press received any report from Secretary Guinn.

